# Maintaining work life under threat of symptoms: a grounded theory study of work life experiences in persons with Irritable Bowel Syndrome

**DOI:** 10.1186/s12876-022-02158-4

**Published:** 2022-02-19

**Authors:** Åsa Frändemark, Hans Törnblom, Magnus Simrén, Sofie Jakobsson

**Affiliations:** 1 Department of Molecular and Clinical Medicine, Institute of Medicine, Sahlgrenska Academy, University of Gothenburg, Gothenburg, Sweden; 2 Centre for Person-Centred Care (GPCC), Sahlgrenska Academy, University of Gothenburg, Gothenburg, Sweden; 3 Institute of Health and Care Sciences, Sahlgrenska Academy, University of Gothenburg, Gothenburg, Sweden

**Keywords:** Irritable bowel syndrome, Work life, Grounded theory

## Abstract

**Background:**

Irritable Bowel Syndrome (IBS) is a highly prevalent functional gastrointestinal disorder. Earlier studies have shown that IBS can limit the ability to perform at work and lead to absenteeism. However, few studies focus on work life experiences based on patients’ narratives. The purpose of this study was to construct a theory for how persons with IBS maintain their work life.

**Methods:**

A qualitative study was performed using constructivist grounded theory. Semi-structured interviews with 15 women and 8 men with IBS (26–64 years of age) were conducted. Fourteen participants worked full-time, six worked part-time and three were on sick leave. The interviews were transcribed verbatim and coded line-by-line, incident-by-incident and thereafter focused coding was done. From the data and codes, categories were generated. Finally, a core category was constructed explaining the process of maintaining work life when living with IBS.

**Results:**

Balancing work life while being under threat of symptoms constituted of four categories, being prepared, restricting impact, reconciling and adjusting. Persons with IBS restricted the impact of IBS on work by using strategies and upholding daily routines and strived to being prepared by exerting control over work life. These ongoing processes served to limit the influence of IBS on work by symptoms being less intense, perceived as less frequent, or not as bothersome. Reconciling IBS with work life was understood as a successful outcome from being prepared and restricting impact but was also influenced by the individual’s outlook on life. Adjusting to other people at work interfered with the strategies of being prepared, restricting impact, and reconciling, leaving persons with IBS more susceptible to symptoms.

**Conclusions:**

This study deepens the understanding of the work situation for persons with IBS. Health care professionals can use the results of this study in the dialogue with the patient discussing work ability and sick leave. The results imply that although balancing work life under threat of symptoms can be a struggle, there are strategies that persons with IBS and employers together can initiate and use to reduce impact on work on several different levels.

## Background

Irritable Bowel Syndrome (IBS) is a highly prevalent functional gastrointestinal disorder characterized by abdominal pain and altered bowel habits [1]. Although being a benign disease from a purely medical point-of-view, IBS impacts multiple aspects of life and is associated with reduced quality of life compared to other chronic diseases and the general population [2, 3]. Having IBS can restrict the possibility to partake in social engagements and relationships [4–6], and can lead to avoidance strategies and adaptive changes in an effort to reduce impact on daily life [5, 7]. Despite these efforts IBS still impacts relationships, self-image, basic functioning, and psychological well-being [5]. Having IBS can also affect how one perceive oneself as a parent or partner [5, 6]. IBS is also associated with several other conditions and symptoms, such as depression, anxiety, fatigue, back pain, and arthralgia [8], and limitations in daily life due to IBS symptoms is greater if having both IBS and psychiatric and other comorbidities [9].

Limitations in daily life due to IBS is not confined to leisure time alone, but also affects work life. Earlier studies have shown that both the number of sick days taken and limitations in the ability to perform well when being at work is increased in subjects with IBS compared to the general population [10, 11] and other chronic diseases such as chronic hand dermatitis [12], and asthma [13]. Having IBS can also influence career choices and some patients report having to change jobs and not pursuing promotions due to their IBS [6, 14]. In a previous study, we have found that work productivity loss was independently associated with IBS symptom severity, general fatigue, and gastrointestinal (GI) symptom-specific anxiety [15]. However, these variables could only explain a small proportion of the work loss in patients with IBS. We have also found that fatigue, another prominent symptom among subjects with IBS, interfered with work and/or studies in a large number of the subjects [16]. Specific GI symptoms frequently reported by IBS patients, such as abdominal pain and bloating, have also been shown to interfere with work and studies [17, 18]. Furthermore, employees with IBS, especially those with diarrhea predominance, are limited by access to toilets [4].

Having chronic conditions can influence work capacity in several ways, e.g. requiring adjustments in the physical work environment, working part-time, or leaving work altogether [19, 20]. In a review published in 2015, de Jong et al. [21] found that job characteristics, social structure and environment, individual work perceptions and organizational characteristics, as well as effects of the disease and treatments contributed to good quality of working life. Although there are some data regarding the impact of IBS on work life, the published studies focused neither on the patients’ experience of work life, nor on factors that help and hinder them at work. Hence, the aim of this study was to construct a theory for how persons with IBS maintain their work life.

## Methods

### Overall study design

Grounded theory (GT) is a method of developing theories based on the data studied, in contrast with methods used to test an existing theory or preconception. GT was first introduced in the late 60’s by Glaser and Strauss [22]. Data is collected and analyzed simultaneously making it possible to obtain answers to new questions throughout the process. In constructivist GT used in this study, introduced by Charmaz in the 90’s, the researcher’s preconceptions, interactions and privileges are part of the social construction that makes up reality [23]. These must therefore be challenged and worked with during the data collection and data analysis process.

### Study population

Participants were recruited from a previously conducted study that focused on the pathophysiology of IBS performed at our secondary/tertiary care outpatient specialist unit at Sahlgrenska University Hospital, Gothenburg, Sweden [24–27]. Eligible for the study were participants with IBS according to the Rome III criteria [28], who were employed and had reported work impairment. Exclusion criteria in the main study were another gastrointestinal diagnosis that could explain the symptoms, severe psychiatric disease as the dominant clinical problem, other severe diseases, and a history of drug or alcohol abuse.

### Data collection

Potential participants received a letter with an invitation to be interviewed. Of the 25 who showed interest, 23 were interviewed individually at one occasion before reaching saturation in accordance with the grounded theory methodology. The interviews were conducted in clusters during two different time points, eight in 2015 and 15 in 2016. The majority of the interviews (n = 17) were jointly conducted by two researchers (SJ and ÅF) and the rest conducted by either SJ (n = 5) and ÅF (n = 1) at our gastroenterology outpatient clinic. The interviewers and respondents met for the first time during the interview that ranged between 23–61 min (median 43 min). The question “Can you tell us about your work situation as it is today?” opened all the interviews and was followed by further open-ended questions aimed at understanding the participants work life in relation to their IBS. In accordance with GT, the data collection and data analysis were made simultaneously [23] making it possible to address topics raised in previous interviews and in concurrent data analysis. To obtain a deeper understanding the interviewers probed the answers by asking “Can you tell me more about [the subject the respondent was talking about]?” and “Can you give an example of [the subject the respondent was talking about]?”. The interviews were conducted in Swedish, tape recorded and transcribed verbatim by the researchers or a third party. Selected quotes were translated to English to illustrate the results.

Demographic information was collected in connection with the interviews. The participants answered validated questionnaires assessing IBS symptom severity (Irritable Bowel Syndrome Severity Scoring System [29]), anxiety and depression (Hospital Anxiety and Depression scale [30]) and general fatigue (Multidimensional Fatigue Inventory 20, general fatigue dimension [31]).

### Data analysis

The qualitative analysis was conducted by the interviewers. In line with constructivist GT memo-writing was used in the analyzing process [23]. As a first step data was coded line-by-line. To further analyze the data from the interviews, the transcripts were entered into Nvivo software (QSR International, 2018), and coded incident by incident. After this focused coding was done. From the data and codes, categories were generated. Where codes are very close to the data, categories were constructed through a degree of conceptual abstraction, while making sure they represent the data. Finally, a core category was constructed. Throughout the analysis process the data, codes and categories were discussed, contrasted, and compared to ensure that they were grounded in the data [32]. Answers from the questionnaires were grouped after severity of symptoms according to recommended cut-offs.

### Ethical considerations

The study was approved by The Regional ethical review board in Gothenburg (diary number 366-15) and followed the ethical principles of the Helsinki declaration. All participants gave their verbal and written informed consent before the start of any study related procedures. Participants were not offered any monetary or other incentives to participate.

## Results

### Participants

Fifteen women and eight men with IBS were included and all participants fulfilled the Rome III criteria for IBS (Table 1) [28]. The majority of participants reported moderate and severe IBS symptoms according to Irritable Bowel Syndrome Severity Scoring System [29]. More than half of the participants reported severe general fatigue [31]. Most participants reported no depression, while anxiety was more prominent with 56 percent reporting possible or probable anxiety according to validated cut-off levels on the Hospital Anxiety and Depression scale [30]. Five participants reported high school and 18 participants reported university as their highest level of education. Most participants worked full-time, about a fourth worked part-time and three participants were on sick leave. The work situation at the time of data collection is shown in Table 2.

**Table 1:** Selected demographics, IBS characteristics and reported symptoms at the time of the interview

Included respondents	
Age, median (range), years	44 (26–64)
Gender, women, n (%)	15 (65)
IBS-SSS	
Mild, n (%)	5 (21.7)
Moderate, n (%)	10 (43.5)
Severe, n (%)	8 (34.8)
MFI general fatigue	
Low, n (%)	4 (17.4)
High, n (%)	7 (30.4)
Severe, n (%)	12 (52.2)
HAD depression	
No, n (%)	16 (69.6)
Possible, n (%)	2 (8.7)
Probable, n (%)	5 (21.7)
HAD anxiety	
No, n (%)	10 (43.5)
Possible, n (%)	4 (17.4)
Probable, n (%)	9 (39.1)

IBS-SSS: Irritable Bowel Syndrome Severity Scoring System, MFI: The Multidimensional Fatigue Inventory 20, HAD: The Hospital Anxiety and Depression scale

**Table 2:** Job characteristics at the time of the interview

Included respondents	
Employment	
Full-time work, n (%)	14 (61)
Part-time work, n (%)	6 (26)
Sick leave, n (%)	3 (13)
Blue-collar workers	5 (21.7)
White-collar workers	18 (78.3)
Occupations^a^, no (%)	
Professionals	9 (39.1)
Technicians and associate professionals	5 (21.7)
Clerical support workers	5 (21.7)
Service, care and sales workers	2 (8.7)
Craft and related trades workers	2 (8.7)

^a^According to Swedish Standard Classification of Occupations 2012, based on International Standard Classification of Occupation 08

### Balancing work life while being under threat of symptoms

**Fig. 1:**
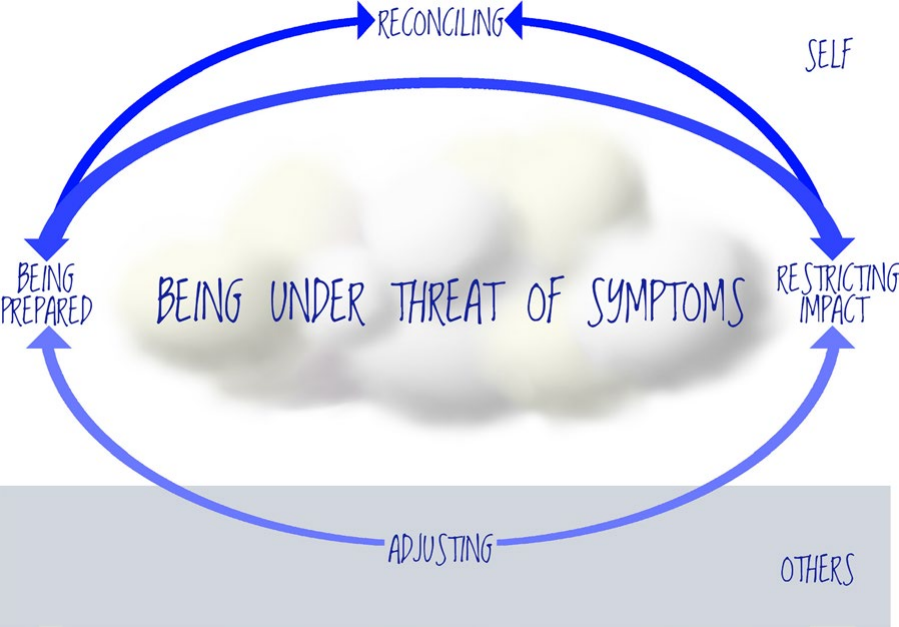
The process of maintaining work life living with irritable bowel syndrome

Balancing work life while being under threat of symptoms formed the core category in this study. The processes of balancing work life comprised of being prepared, restricting impact, reconciling, and adjusting. All these processes revolved around being under threat of symptoms visualized in Fig. 1. Being prepared, restricting impact, and reconciling, were interpreted as being dependent on oneself. Persons with IBS use strategies and routines and exert different forms of control to be prepared and restrict impact of IBS on work. Reconciliation of work and IBS was understood as a successful outcome from being prepared and restricting impact, and was influenced by outlook on life. In reconciliation, the impact of IBS on work was described as decreased, the symptoms were perceived as less frequent, less intense and/or not as bothersome. In contrast, adjusting to others and thereby not being able to take full advantage of being prepared and restricting impact leads to additional challenges in work life and a greater impact from being under the threat of symptoms. In Table 3, categories are exemplified with quotes from the interviews.

**Table 3:** Quotes from the interviews regarding the process of maintaining work life living with irritable bowel syndrome

Process	Quotes
Being prepared	“I’ve done that because I’m tired. […] I do not have the energy to work full-time. If that has to do with the gut or not is very hard to know”
	” If I feel something coming on, I try to swap tasks with someone so that I have the opportunity to hurry away. Because the problem is when I can’t leave when I have to”
	“Now I have the possibility to work from home. And I do not use that opportunity very often, but it’s possible. So that is always… It feels good to have that possibility. If it should be needed. Then you have a toilet close by and you can be on your own, so that works quite well”
	“I try to keep myself updated about if there’s any fun jobs available. But then I think “no, it’s not a good fit for me” […] I try to adjust after my ability”
Restricting impact	“It has affected me a lot, when I’ve traveled as part of my work, I’ve had to submit special need of diet and preordered food, at conferences and other meetings. And since a couple of years back I always bring a lunch box to work to know exactly what I eat”
	“I’ve seen a nurse that works with hypnosis, with the gut in focus. […] I have worked with relaxation exercises for quite some time, so she says it is easy for me to go in and out of the hypnotic states or what they are called. So, I do yoga as well. Breathing, and then of course prayer as a tool for me as a Christian”
	“The more you sit still, the worse the gut symptoms”
	“We are three people who sit together and when we have talked it has been evident that we have different problems, and one of them is now about to go through examinations that you’ve been through yourself. So, it’s quite open discussions in that group”
	“They don’t need to see what I am doing all the time. I do not need that extra ordeal. That I have to tell exactly where I am and that now I have to rush to the toilet”
Reconciling	“Once I had figured out what I had, what I could eat and what I could not eat, I just make sure to stick to that. Of course, I still have pain sometimes, but I have learnt to live with it. So, I do not think it affects me as much. Especially not in work life”
	“It can be a struggle, but you have to keep in mind *What is the alternative?* What else could I do? And would that really make things substantially better? I’m not convinced”
	“It’s not something I think anyone should feel bad about. It is not up to me to be well. You either are or you are not. I think I perform damned well anyways”
	“I have had to learn that it is OK to make mistakes […] It doesn’t matter; I don’t work as a neurosurgeon or a pilot. It does not have any major consequences. If it does and someone gets angry, then that is the way that is”
	“It would have been easier for the gut and everything to get an employment and just work. But that is not fun, so the head does not want that, so I listen more to the head than to the gut”
	“I have understood that many people can’t work at all. So, then you can say that I am fortunate”
Adjusting	“But there have been many times where I have had to go home from work, especially when we’ve been two working [together]. Not because I have to go to the toilet but mostly because of pain. It gets even worse in some way”
	“You have to relate to people all the time and how they think and react, and it can be hard. When you are tired, and in pain, you cannot really turn that off. […] The pain”
	“But it’s not like I would stay at home an entire day; I won’t do that. Now that I have children it’s almost calmer at work than at home”

### Being under threat of symptoms

The unpredictable characters of symptoms affected work life. The respondents spend a lot of time being perceptive to their gut, thinking and worrying about present symptoms and possible worsening of symptoms. Symptoms from the GI tract and fatigue were specific symptoms, which were described as interfering with work life. GI symptoms delayed arriving in time in the mornings and disrupted and hindered work tasks during the day due to urgent need to visit the toilet. For many, the GI symptoms could flare up, which led to a period with intense symptoms from their IBS. During these periods, which could vary from one day to weeks, working was almost impossible. Fatigue was described by many as impacting work life more than GI symptoms. Multiple aspects of work were affected by mental and/or physical fatigue, making it harder to concentrate and affecting the pace of work and could even make full-time work impossible. Respondents also described co-occurrence of fatigue and pain, where pain could lead to fatigue, requiring rest, and depending on severity, a need to stay home from work. Stress from having symptoms, as well as stress originating from work life led to more symptoms. The stress did not always trigger symptoms but could intensify already occurring symptoms. Experiencing anxiety could trigger symptoms.

### Being prepared

Being prepared was understood as strategies to prevent or decrease symptom burden at work. Being prepared was done by controlling and/or adapting different aspects of work life. However, controlling work life did not always mean having control as other parts of life interfered. Moreover, not having a permanent employment could reduce the possibility of having control in work life.

One strategy of being prepared was the possibility to control work time and work hours; be able to choose to work part-time, take advantage of flexible hours, and have the freedom to schedule time at work yourself. The possibility to control time by arriving to work later and staying longer or arrive earlier another day could prevent an entire day of sick leave due to symptoms. Controlling time when being at work involved scheduling meetings and trips at times when the symptom burden was not too severe. Work life took a lot of energy, and to work part-time was described as a way of preserving energy for private life. To vary work tasks gave the respondents an opportunity to choose suitable tasks in relation to symptoms. Having the option to work on your own, with tasks that could be left at any time was useful at times when experiencing GI symptoms. Work tasks that had critical steps that could not be interrupted and tasks with higher demands was chosen when GI symptoms were absent. Being able to control the place of work, from home, at the office or outside, was described as another way of being prepared and thereby being able to prevent or decrease the symptom burden. The possibility to work from home, and having access to a toilet, could allow respondents to work despite having GI symptoms. Controlling the place of work also included being aware of where the nearest toilet is when choosing workstation and planning toilet breaks during work hours.

Being prepared also involved choosing occupations and careers that were compatible with IBS. This took the following expressions: reservations in choosing professions that demands working close to others, pursuing work that allows you to maintain routines, or hesitating to seek jobs out of fear of not having the energy to take on a new professional role.

### Restricting impact

Restricting impact of IBS on work life was interpreted as an ongoing process involving practical strategies that took up a lot of time and thought. Strategies involved maintaining routines that helped to reduce the symptom burden or restrict the impact of them. Not being able to uphold strategies and routines was described as stressful, which was especially difficult to do when traveling was part of your work.

Controlling food intake during work hours was prominent. Respondents brought food from home for lunch, choose to buy food at lunch restaurants that was compatible with their dietary choices or restricted the amount of food they ate when at work. Choosing safe food when traveling as part of work, both during and outside of work hours, was also described as a strategy. Apart from strategies concerning food intake, strategies and routines around physical activity, relaxation and preparation was evident. By listening to relaxation tapes, using strategies learnt from hypnotherapy and using breathing exercises stressful work situations could be relieved. Respondents also used these strategies, as well as yoga and prayer, in their spare time in order to reduce impact of stress on overall life. Physical activity was used to control GI symptoms. Exercising regularly and avoiding long periods of sedentary work tasks was considered important. Having the opportunity to stand up and work was considered positive.

Respondents restricted impact by preparing for work by using routines in relation to their gut. This was done by waking up early and having plenty of time for bowel movements, not drinking coffee before arriving at work or packing extra underwear and toilet paper or tissues to have close by in case of an GI-related accident. Another way to restrict impact of IBS was to seek support from others at work. Colleagues and/or the employer could be a source of support in providing an opportunity to share experiences of illness. Asking colleagues for support could ease the work situation, increase productivity, and job satisfaction. However, all respondents did not get support from colleagues and some felt monitored when for example visiting the toilet. In some cases, loneliness was described, since others did not understand what living with IBS entailed and respondents feared reactions from colleagues noticing them passing gas or thinking of them as being lazy. On the other hand, restricting impact of IBS on work could also result in choosing solitude. Having an office of your own, with the possibility to close the door and to be alone was of importance for some. Choosing solitude had the benefit of making work feel more efficient. Further, when experiencing symptoms such as pain, it was easier to be able to continue to work on your own and not having to interact with other people. Working on your own was also something that was described as positive regarding being able to go to the toilet without colleagues noticing this.

### Reconciling

Reconciliation of work life and IBS occurred on different levels. One more superficial level was when being prepared and restricting impact worked well and the threat of symptoms lessened at work. Another, more profound level, comprised a basic positive outlook on life and work life, which was described as important to lessen the burden of IBS at work. Having a general positive outlook on life was an aspect of reconciling, which helped some to uphold their work life. For example, having to eat often created natural, much needed, minibreaks from work. Seeing one’s abdomen as part of a whole, with body, mind, and spirit, and focusing on what that whole is capable of at work, helped coming to terms with IBS. Some compared their situation to others, who had other or worse IBS symptoms and could find feelings of gratefulness over their own situation.

Expanding knowledge of IBS was a lifelong endeavor and included learning to listen to the gut and finding strategies that could help reconcile IBS and work life. Expanding knowledge was done by a combination of listening to the gut, searching for information and trial-and-error. For example, respondents could over time learn to recognize different types of pain, making a familiar pain less frightening based on previous experiences. Finding strategies was something respondents had to do on their own, since many respondents felt they had not received any help from health care professionals. However, multidisciplinary group education and hypnotherapy given at a specialist clinic were brought forward as being helpful.

Several respondents chose not to be hindered by their IBS, chose not to surrender to it, or expressed that they decided to not be ashamed of living and working with IBS. Since the illness was not their choice, they did not see it as their fault if they now and then struggled with work tasks, which could be compensated for when the symptom burden was less severe. Some of the respondents reported having been brought up not to make a fuss and therefore had learnt to endure when experiencing symptoms at work. Because of this they were not that affected by IBS at work anymore, which resulted in no need to tell colleagues about their IBS. Closely related to not surrendering was coming to terms with the IBS diagnosis and accepting the situation after having had IBS for many years. When the respondent liked their work, and felt that they were doing something important, that could be chosen over a job that was more compatible with IBS symptoms. This was done because the job satisfaction was highly valued and outweighed the potential benefit of decreased severity of IBS symptoms. This also gave strength to work through symptoms.

The workplace was sometimes seen as a safe haven. Working reduced the impact of IBS by diverting attention from the gut. Having access to a resting room gave a sense of safety and could reduce symptoms, even if seldom using it. Not being subjected to the same expectations when it came to looks and appearance at work as in social life was seen as something positive. Depending on workplace, wearing functional work clothes, that was more comfortable for the abdomen than regular clothes, could be preferred.

### Adjusting

Adjusting and relating to others was often described as being negative. When adjusting, respondents could not fully take advantage of being prepared and restricting impact. The threat of symptoms was magnified, and symptoms broke through more often impacting the work situation. Having to adjust to colleagues could mean not being able to choose work tasks that were suitable for the gut or eating food that was not good for them. It was often seen as stressful to work close to colleagues, which could worsen IBS symptoms. Sharing a room or working close to colleagues also caused symptoms directly, e.g., having long meetings and not being able to pass gas could lead to abdominal pain. Some noticed that they had less severe IBS symptoms when they were off work since they did not have to adjust to others and some reported that symptoms decreased when they simply stopped adjusting to others at work.

To relate to others emotionally as part of specific work tasks was also something that made respondents more exposed to IBS symptoms. Working towards another person, e.g., in social work, with high demands on being attentive and empathic, took a lot of mental energy and left respondents more vulnerable to symptoms. This did not only apply to social work, but also working with patients, clients, and some costumers. Some participants reported experiencing ethical stress in their work, which was described as an additional factor causing stress. Respondents who did not have close contact with other people as part of their work tasks expressed that they preferred to work when experiencing distressful symptoms as adjusting and relating to other people also applied to family life.

## Discussion

This study used constructivist grounded theory to explore work life in persons with IBS. This is, to the best of our knowledge, the first study to construct a theoretical model for the process of maintaining work life for persons with IBS. Our model suggests that maintaining work life is an ongoing process that revolves around a constant threat of symptoms where strategies, daily routines, control over work life and outlook on life can decrease the threat, while having to adjust to others can hinder the preventative processes of being prepared and restricting impact and therefore lead to an increased susceptibility to symptoms. Enhanced understanding of work life in subjects with IBS is clearly needed based on the high proportion of subjects with IBS with reduced work productivity and activity [15].

Perhaps the most prominent aspect expressed in the interviews was the focus of the respondents on symptoms and the gut and a constant focus on being prepared, restricting impact and reconciliation. Many respondents described their IBS as being unpredictable with periods with no or mild symptoms, and sudden flare-ups with severe symptoms lasting for a few days to weeks. Even if the symptoms were not present, the respondents were aware of the gut and signs of possible symptoms. They registered sounds, sensations, the effects of stress and food intake. Further, they planned and prepared for current and emerging symptoms, in relation to work. Being perceptive of the body and symptoms during work have been reported in studies with persons with other chronic diseases, and the focus seems to differ somewhat depending on the disease. In a study by Hjarsbech et al. [33], employees with depressive symptoms listened to signals from the body to reduce negative emotions and symptoms. Löfgren et al. [34] studied work life in women with fibromyalgia and described how the informants were perceptive of their bodies by using pain as a guide. By doing this they could either prevent worsening of symptoms by taking a break from what they were doing or chose to endure if the task at hand was prioritized over the symptoms [34]. In a study by Boot et al. [35], the subgroup of employees with chronic obstructive pulmonary disease described being preoccupied with their health being affected by respiratory symptoms, and this could be frightening all the time. Consequently, focusing on the gut is something that differs IBS from other chronic diseases when it comes to work. However, the consequences of focusing on symptoms seems to be similar for diseases sharing certain characteristics, such as variation in symptoms over time.

One aspect of work life that our study highlights is the respondents’ determination to work. Working was expressed as something positive, adding something more than merely an income. Having a work to focus on provided distraction from IBS symptoms. Not surrendering to IBS, by not letting oneself be defined by IBS and learning to work through distressful symptoms could reduce the impact on work. The importance of working from a social perspective, and of job satisfaction for well-being has been known for long [36], and this has also been emphasized in previous studies examining work in other long-term conditions, such as rheumatoid arthritis [37], depressive symptoms [33] and fibromyalgia [38]. The respondents in this study did not only work, but they also worked hard on maintaining their work life. Furthermore, the respondents were ambitious when it came to their careers, either being satisfied with their current positions or searching for new job opportunities that they found more interesting or that would suit them better. Changing careers after being well-established at the job market was not described, but in some cases, IBS influenced the choice of education. By comparison, a study by Palstam et al. [38] reported that changing career after diagnosis was one way to promote work sustainability for women with fibromyalgia. This difference might be due to the early onset of IBS, where most of our respondents had suffered from IBS since adolescence and therefore had had time to develop strategies and routines and thus found a way to balance work life and IBS. Taking sick-leave one day because of symptoms was rare since staying at home did not help with the symptoms and they did not want to neglect work.

As our results show, seeking support from colleagues was one way to restrict the impact of IBS on work. The importance of the social work environment, including both colleagues and upper management has been reported for workers with other chronic diseases than IBS [21, 33, 35, 39]. However, for some respondents support from colleagues was not always present. Lack of support could mean that colleagues did not understand the importance for the respondents of e.g. upholding routines around food intake. This is in line with some previous studies on other chronic illnesses and work [35, 40]. In our model, adjusting to others was found to be an obstacle for the respondents in work life. This obstacle was both described in interactions with colleagues, and in interactions with clients, patients, or customers. This is in line with findings in a study by Bertilsson et al. [40] on employees with common mental disorders, where interpersonal encounters at work was the work tasks described as most demanding. However, the participants in that study described interpersonal encounters as hard due to loss of empathy and emotional detachment [40], which was not found in the respondents in our study, suggesting different mechanisms in persons with IBS and common mental disorders.

Fatigue is a common and distressing symptom in patients with IBS, even though it is not part of the diagnostic criteria for IBS. We have previously demonstrated that it affects life in a multidimensional way, with poor bodily stamina being the most prominent feature [16]. In this study, fatigue was described by many of the subjects as affecting work life more than GI symptoms. Multiple aspects of work were affected by mental and/or physical fatigue, making it harder to concentrate and affecting the pace of work and could even make full-time work impossible. This highlights the fact that management of patients with IBS cannot only focus on treating GI symptoms, but a holistic approach is needed, including management of fatigue and other comorbid symptoms, such as non-GI somatic symptoms and psychological factors [41].

A qualitative methodology was used as we wanted to be able to explore the patient’s perspective without the restrictions that e.g. questionnaires have. Grounded theory was suitable as we wanted to explore action and strategies to deepen the understanding of a relatively novel area in patients with IBS. When it comes to the study sample, the respondents were mostly white-collar workers with a high educational level. Most of the respondents in our study were female, born in Sweden and the study took place in Sweden’s second largest city, which might explain why 78% had studied beyond high school. There was also a dominance of respondents with moderate and severe IBS. However, we argue that the qualitative methodology of the study and achieving saturation in the data by a thorough analysis and conclusion of the characteristics of the categories grounded in data, increases the usefulness and validity of the results of the study.

## Conclusions

To conclude, this study deepens the understanding of work life of persons with IBS and the impact of IBS on work. Health care professionals can use the results of this study when meeting patients with IBS and when discussing IBS in relation to work ability and sick leave. Our results imply that although balancing work life with living with IBS can be a struggle, there are ways for persons with IBS to reduce the impact on work on several different levels. The strategies described in this article can be used as a starting point for discussion in different forms of patient education.

## Data Availability

The datasets used and/or analyses during the current study are available from the corresponding author on reasonable request.

## Abbreviations

IBS: Irritable bowel syndrome
GT: Grounded theory
GI: Gastrointestinal
COPD: Chronic obstructive pulmonary disease
